# Comparative Genome Analysis Reveals Differences in Biocontrol Efficacy According to Each Individual Isolate Belonging to Rhizospheric Fluorescent Pseudomonads

**DOI:** 10.1264/jsme2.ME21034

**Published:** 2021-07-29

**Authors:** Ryohei Sakuraoka, Nobutaka Someya, Kasumi Takeuchi, Tomohiro Suzuki, Tomohiro Morohoshi

**Affiliations:** 1 Department of Innovation Systems Engineering, Graduate School of Engineering, Utsunomiya University, 7–1–2 Yoto, Utsunomiya 321–8585, Japan; 2 Institute for Plant Protection, National Agriculture and Food Research Organization (NARO), 2–1–18 Kannondai, Tsukuba, Ibaraki 305–8666; 3 Institute of Agrobiological Sciences, NARO, 2–1–2 Kannondai, Tsukuba, Ibaraki 305–8602, Japan; 4 Center for Bioscience Research and Education, Utsunomiya University, 350 Mine-machi, Utsunomiya, Tochigi 321–8505, Japan

**Keywords:** fluorescent pseudomonads, biocontrol, antibiotic, comparative genome analysis, complete genome sequencing

## Abstract

Biocontrol fluorescent pseudomonads produce a number of antibiotic organic compounds, including 2,4-diacetylphloroglucinol, pyoluteorin, pyrrolnitrin, and phenazine. We previously classified rhizospheric fluorescent pseudomonads harboring antibiotic biosynthetic gene clusters into 10 operational taxonomic units (OTUs). In the present study, we report the complete genome sequences of selected strains from these OTUs. The genetic diversity of antibiotic biosynthetic gene clusters and their surrounding sequences correlated with the OTU classification. In comparisons of the biocontrol activity and distribution of antibiotic biosynthetic gene clusters, we found that the pyrrolnitrin biosynthetic gene cluster more effectively controlled the growth of *Rhizoctonia solani*.

*Pseudomonas* is one of the most diversified bacterial genera and is widespread in multiple environments (Peix *et al.*, 2009). Fluorescent pseudomonads are a specific group of the genus *Pseudomonas* that produce an extracellular, water-soluble, yellow-green pigment that fluoresces under UV irradiation (Bultreys *et al.*, 2003). Several fluorescent pseudomonads produce antibiotics, exhibit biocontrol activities, and protect host plants (Höfte and Altier, 2010). These biocontrol fluorescent pseudomonads produce a number of antibiotic organic compounds, such as 2,4-diacetylphloroglucinol (PHL), pyoluteorin (PLT), pyrrolnitrin (PRN), and phenazine (PHZ) (Gross and Loper, 2009), which exhibit effective biocontrol activities against phytopathogens within their respective antimicrobial spectra (Howell and Stipanovic, 1979, 1980; He *et al.*, 2004). In contrast, the diversity of fluorescent pseudomonads and the distribution of antibiotic biosynthesis genes have not yet been clearly elucidated. We previously isolated more than 3,000 strains of fluorescent pseudomonads from plant roots in Japan and screened for the presence of antibiotic-synthesizing genes (Someya *et al.*, 2020). A cluster analysis based on 16S rRNA sequences revealed that fluorescent pseudomonads may be classified into 10 operational taxonomic units (OTUs). When the presence of antibiotic biosynthesis genes was assessed via PCR with specific primers, OTU HLR comprised three antibiotic biosynthesis genes (PHL, PLT, and PRN), OTU RZ contained two antibiotic biosynthesis genes (PRN and PHZ), and OTU H1, H2, H3, H4, H5, H6, H7, and H8 comprised only one antibiotic biosynthesis gene (PHL); however, when the biocontrol efficacy of each OTU was examined using a cabbage-*Rhizoctonia solani* pathosystem, the OTU classification and possession of antibiotic biosynthesis genes did not show a clear relationship with biocontrol efficacy (Someya *et al.*, 2020). Based on these findings, we hypothesized that the genetic diversity of antibiotic biosynthetic gene clusters in the genome of fluorescent pseudomonads may be closely related to the production of antibiotics and biocontrol efficacy. In the present study, we report the complete genome sequences of 13 strains selected from the aforementioned 10 OTUs, clarify the distribution of antibiotic biosynthetic gene clusters in their genome, and reveal the relationships among the diversity of antibiotic biosynthetic gene clusters, production of antibiotic compounds, and biocontrol efficacy.

The 13 strains used in the complete genome analysis are listed in Table 1. The complete genome sequences of three strains, Cab57, Os17, and St29, were elucidated in our previous study (Takeuchi *et al.*, 2014; 2015). Regarding the genome sequencing of the other 10 strains, bacterial strains were cultured in trypticase soy broth (TSB; Becton, Dickinson and Company) at 30°C with shaking. Total genomic DNA was extracted using NucleoSpin Tissue (Takara Bio), according to the manufacturer’s protocol. Genome sequencing was performed on the PacBio RSII platform (Pacific Biosciences) using libraries prepared with SMRTbell Template Prep Kit 1.0 (Pacific Biosciences) by Macrogen Japan. Sequencing reads were assembled using Canu version 1.9 (Koren *et al.*, 2017). Circular contigs were subjected to the prediction and annotation of genes using DFAST pipeline 1.2.11 (Tanizawa *et al.*, 2018). Coding sequences (CDSs) were predicted using Prodigal 2.6.3 (Hyatt *et al.*, 2010). Genes coding for tRNA and rRNA were discovered using Aragorn 1.2.38 (Laslett and Canback, 2004) and Barrnap 0.8 (https://github.com/tseemann/barrnap), respectively. A homology search was conducted using BLAST software (Altschul *et al.*, 1990) in SequenceServer 1.0.14 (Priyam *et al.*, 2019). The phylogenetic tree based on whole genome alignments was constructed using REALPHY 1.12 (Bertels *et al.*, 2014). The general features and DDBJ/ENA/GenBank accession numbers of the complete genome sequences of the *Pseudomonas* strains used in the present study are described in Table 1.

We assessed the presence of antibiotic biosynthetic gene clusters in the complete genome of 13 strains. A phylogenetic tree based on the complete sequences of the chromosome of *Pseudomonas* strains and the presence of an antibiotic biosynthesis gene cluster is shown in Fig. S1. The *phl* gene cluster for PHL biosynthesis, which consists of nine genes, was present in all complete genome sequences, except for Tre132 from OTU RZ. The *phl* gene cluster was widely distributed in *Pseudomonas protegens*, *Pseudomonas saponiphila*, *Pseudomonas fluorescens*, and *Pseudomonas brassicacearum*, which comprised OTU HLR, H1, H2, H3, and H4 (Fig. S1). We compared the sequences of the *phl* gene cluster and its surrounding regions in the complete genome sequences of these strains (Fig. 1). The sequences surrounding the *phl* gene cluster from OTU HLR and H1 were almost identical. The upstream sequences of the *phl* gene cluster of OTUs H2, H3, and H4 were very similar, and the downstream sequences were closely related to the phylogenetic relationship described in Fig. S1. The sequence of the *phl* gene cluster and its surrounding region in Cab53 from OTU H5 and its related strain *Pseudomonas chlororaphis* UFB2 indicated high similarity; however, the complete genome sequence of UFB2 markedly differed from that of *P. chlororaphis* subsp. *aurantiaca* type strain DSM 19603^T^, which did not possess the *phl* gene cluster (Fig. 1). These results showed that UFB2 and Cab53 may be more accurately classified as other *Pseudomonas* species and not *P. chlororaphis*. The complete genome sequences of *Pseudomonas baetica* a390^T^, *Pseudomonas agarici* LMG 2112^T^, and *Pseudomonas alcaligenes* NBRC 14159^T^, which were phylogenetically related to Seg1 from OTU H6, Ost2 from OTU H7, and Pc102 from OTU H8, respectively, did not contain the *phl* gene cluster. The downstream sequences of the *phl* gene cluster of Ost2 were only detected in the genome of *P. chlororaphis* PCL1606, which did not possess the *phl* gene cluster. Furthermore, the surrounding sequence of the *phl* gene cluster in Seg1 and PC102 was not found in any sequences deposited in the DDBJ/ENA/GenBank databases. Based on these results, the *phl* gene cluster did not appear to be originally equipped in OTU H6, H7, and H8, and was presumably acquired due to the introduction of another large DNA molecule into their genome by horizontal gene transfer.

To evaluate the relationship between the diversity of the *phl* gene cluster and production of PHL, we assessed PHL production by 31 strains from the *phl* gene cluster-positive OTUs described in a previous study (Someya *et al.*, 2020) (Table S1). To extract PHL from the culture supernatant, bacterial strains were inoculated into 4‍ ‍mL of nutrient broth (Becton, Dickinson and Company) containing 1% (w/v) glucose (NBGlu) medium. After an incubation for 18 h, bacterial cultures were inoculated into 4‍ ‍mL of fresh NBGlu medium (1% inoculum) and incubated for a further 16‍ ‍h. Thereafter, culture supernatants (200‍ ‍μL) were mixed with an equal volume of acetonitrile. Samples (20‍ ‍μL) were chromatographed on an HPLC system (Jasco) with a UV/VIS detector set at 270‍ ‍nm using a Mightysil RP-18GP column (250×4.6‍ ‍mm, particle diameter of 5‍ ‍μm; Kanto Kagaku). Samples were eluted isocratically with water:acetonitrile:acetic acid (50:50:0.1 [v/v/v]) at 2‍ ‍mL min^–1^. The amount of PHL was estimated by comparing the peak areas for a given retention time with respect to the PHL solution of a known concentration. The results of the PHL quantification are shown in Fig. S2. The nine strains of OTU H2 showed high production levels of PHL, while six strains of OTUs H4, H5, and H6 showed moderate production levels of PHL. In contrast, the other 15 strains belonging to OTU HLR, H1, H3, H7, and H8 exhibited low production levels of PHL. Although the complete genome sequence and surrounding sequence of the *phl* gene cluster correlated among OTUs H2, H3, and H4 (Fig. 1 and S1), a marked difference was observed in PHL production levels among these three OTUs. Differences in the regulatory system for the expression of the *phl* gene cluster was assumed to have affected PHL production in the three OTUs.

The *prn* gene cluster for PRN biosynthesis, which comprises four genes, was only present in the complete genome sequences of OTU HLR and RZ, whereas Eqa60, belonging to OTU HLZ, did not possess this gene cluster. We compared the *prn* gene cluster and its surrounding sequence in the complete genome sequence of the OTU HLR strains, Cab57, Boi14, and Eqa60 and *P. protegens* type strain CHA0^T^ (Fig. 2A), and found that the sequence of the *prn* gene cluster and its surrounding sequences, CHA0, Cab57, and Boi14, were similar. In the genome sequence of Eqa60, a 16-kbp region containing the *prn* gene cluster was deleted, and a 6-kbp sequence containing a putative transposon gene was inserted (Fig. 2A). The *prn* gene cluster was presumable eliminated due to the insertion of a transposon in the genome of Eqa60. The *plt* gene cluster, which consists of 17 genes, was only present in the complete genome sequence of OTU HLR. The sequence of the *plt* gene cluster and its surrounding sequence in CHA0, Cab57, and Eqa60 were well conserved (Fig. 2B). In contrast, Boi14 belonging to OTU HLR did not possess the *plt* gene cluster in its genome, and a 36-kbp region containing the *plt* gene cluster, which was conserved in other OTU HLR strains, was completely deleted in the genome of Boi14 (Fig. 2B). The *phz* gene cluster for PHZ biosynthesis, which consists of 10 genes, was only present in the complete genome sequence OTU RZ. The sequence of the *phz* gene cluster was similar between Tre132 and *P. chlororaphis* subsp. *aurantiaca* type strain DSM 19603^T^ (data not shown).

We previously assessed the biocontrol activities of the 13 complete genome-sequenced strains examined in the present study using a cabbage-*R. solani* pathosystem (Someya *et al.*, 2020). The findings obtained demonstrated that most strains of OTU HLR, RZ, and H7 exhibited higher biocontrol activities (Table 1). Although PHL is an antibiotic produced by OTU HLR and H7 strains, it was produced in limited quantities by OTU HLR and H7 (Fig. S2). In contrast, although OTU H2 and H5 strains showed higher production levels of PHL, they did not exhibit any significant biocontrol activity in this system. PHL plays a pivotal role in the biological control of diseases caused by *R. solani* (He *et al.*, 2004). The present results suggest that not all strains harboring the *phl* gene cluster effectively produce PHL on the plant surface; however, the activation of the *phl* gene cluster may be necessary to enhance the biocontrol activity of the strains that affect PHL production. The OTUs HLR and RZ, which exhibited higher biocontrol activities, share the *prn* gene cluster. Moreover, PRN was shown to exert inhibitory effects against diseases caused by *R. solani* (Cartwright *et al.*, 1995). Most strains belonging to OTU HLR exhibited high biocontrol activities, with Boi14 showing the highest biocontrol activity among all strains used in this study (Someya *et al.*, 2020). In contrast, one of the HLR strains, Eqa60, did not exhibit any biocontrol activity (Table 1). The comparative genome analysis performed in this study revealed that Eqa60 and Boi14 lacked the *prn* and *plt* gene clusters, respectively, in their genomes. These results suggest that the production of PRN rather than PLT is important for biocontrol activity against diseases caused by *R. solani*. In a future study, a mutation analysis of each cluster will reveal their functional relevance. As an exception, Ost2 from OTU H7, which did not possess the *prn* gene cluster, exhibited slight biocontrol activity, indicating that unknown genes involved in biocontrol activity are encoded in the genome of Ost2.

In conclusion, we herein demonstrated a relationship between the distribution of antibiotic biosynthetic gene clusters and their biocontrol efficacy through the complete genome sequencing of rhizospheric fluorescent pseudomonads. The present results suggest that PRN is the most effective antibiotic in the control of diseases caused by *R. solani*. Regarding other antibiotics, particularly PHL, the expression of antibiotic biosynthesis gene clusters may not be effectively performed on the surface of plants because some strains harboring the *phl* gene cluster did not exhibit biocontrol activity using the cabbage-*R. solani* pathosystem, but showed higher production levels of PHL under our experimental conditions. A previous study reported that the efficacy of biological control differed between controlled and commercial field conditions, and was less effective or completely ineffective when introduced under the latter conditions (Guetsky *et al.*, 2001). To develop more effective biocontrol agents, further studies need to investigate not only on the presence of antibiotic biosynthesis gene clusters in the genome, but also attempt to activate and stabilize the expression of these gene clusters under uncontrolled field conditions.

## Citation

Sakuraoka, R., Someya, N., Takeuchi, K., Suzuki, T., and Morohoshi, T. (2021) Comparative Genome Analysis Reveals Differences in Biocontrol Efficacy According to Each Individual Isolate Belonging to Rhizospheric Fluorescent Pseudomonads. *Microbes Environ ***36**: ME21034.

https://doi.org/10.1264/jsme2.ME21034

## Supplementary Material

Supplementary Material

## Figures and Tables

**Fig. 1.**
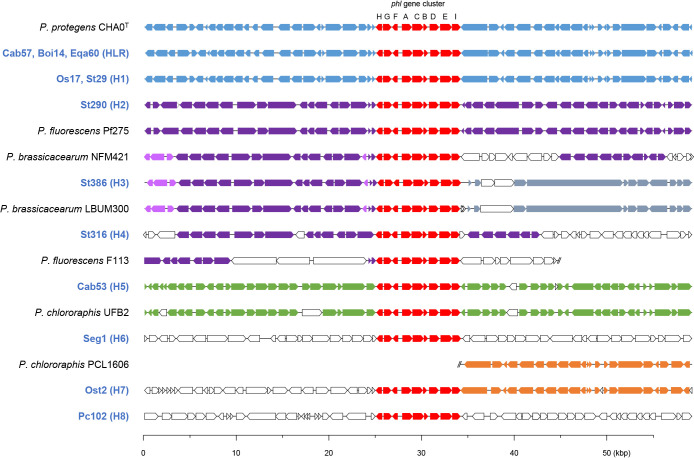
The arrangement of genes around the *phl* gene cluster. Genes and their orientations are depicted with arrows. The *phl* gene cluster is colored in red. Genes of homologous synteny are shown using the same color. Other genes without homologous synteny are shown in white. Strains with genomes that were sequenced in our present or previous study are shown in blue. The scale represents a 50-kb length of nucleotides.

**Fig. 2.**
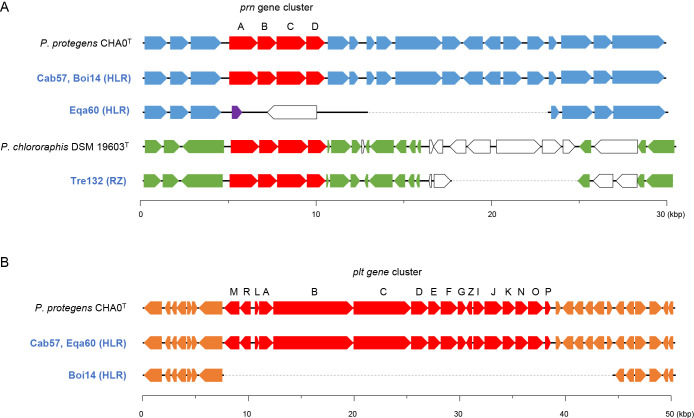
The arrangement of genes around the *prn* (A) or *plt* (B) gene cluster. Genes and their orientations are depicted with arrows. The *prn* or *plt* gene cluster is colored in red. The putative transposon gene is colored in purple. Genes of homologous synteny are shown using the same color. Other genes without homologous synteny are shown in white. Strains with genomes that were sequenced in our present or previous study are shown in blue. The scale bar represents a 30-kb (A) or 50-kb (B) length of nucleotides.

**Table 1.** Summary of general features of the complete genome of *Pseudomonas* strains used in the present study.

Strain	OTU	Biocontrol activity^a^	Genome information						
			Size (bp)	CDS	rRNA	tRNA	GC%	Acc. no.	Reference
Cab57	HLR	++	6,827,892	6,186	16	68	63.3	AP014522	Takeuchi *et al.*, 2014
Boi14	HLR	+++	6,968,016	7,760	16	75	63.4	AP024341	This study
Eqa60	HLR	–	6,945,474	6,306	16	74	63.3	AP024342	This study
Tre132	RZ	+++	6,961,699	6,519	16	73	62.8	AP020336	This study
Os17	H1	–	6,885,464	6,284	19	70	63.5	AP014627	Takeuchi *et al.*, 2015
St29	H1	–	6,833,117	6,102	19	68	63.3	AP014628	Takeuchi *et al.*, 2015
St290	H2	–	6,775,898	5,892	15	65	60.8	AP014703	This study
St386	H3	–	6,821,346	6,036	16	67	60.8	AP021900	This study
St316	H4	–	6,777,545	6,049	16	69	60.5	AP021901	This study
Cab53	H5	–	6,259,290	5,537	16	71	62.2	AP021902	This study
Seg1	H6	–	6,625,535	5,900	19	71	59.2	AP021903	This study
Ost2	H7	+	7,357,961	6,462	19	79	62.9	AP021904	This study
Pc102	H8	–	6,682,531	5,990	12	69	66.8	AP021905	This study

^a^ For biocontrol activities against cabbage damping off caused by *Rhizoctonia solani*, previous findings (Someya *et al.*, 2020) were used with modifications. +++, <75% inhibition; ++, 50–75% inhibition; +, 25–50% inhibition; –, 0–25% inhibition.
