# Supplementary material for: Comparative Genome Analysis Reveals Differences in Biocontrol Efficacy According to Each Individual Isolate Belonging to Rhizospheric Fluorescent Pseudomonads
Source: Microbes Environ. 2021 Jul 29;36(3):ME21034. doi: 10.1264/jsme2.ME21034 (PMC8446755; doi:10.1264/jsme2.ME21034)
Supplement: Supplementary file 1 — Supplementary Material [file 36_21034_s1.pdf]

Table S1. General information of *Pseudomonas* strains used in this study

OTU	Strain	Host	Collection site	Biocontrol activity[a]
HLR	Boi14	*Brassica oleracea* var. *italica*	Koga, Ibaraki, Japan	+++
HLR	Cab57	*Capsella bursa-pastoris* (L.) Medik.	Shikaoi, Hokkaido, Japan	++
HLR	Eqa60	*Equisetum arvense* L.	Monbetsu, Hokkaido, Japan	–
HLR	Pan63	*Poa annua* L.	Ryugasaki, Ibaraki, Japan	++
HLR	Pc101	*Phaseolus coccineus* L.	Tsukuba, Ibaraki, Japan	++
HLR	Tan3	*Tylophora aristolochioides* Miq.	Fujimi, Nagano, Japan	+++
HLR	Tre92	*Trifolium repens* L.	Takahagi, Ibaraki, Japan	+++
RZ	Tre132	*Trifolium repens* L.	Tsukuba, Ibaraki, Japan	+++
H1	Arp28	*Artemisia princeps* Pampan.	Hitachi, Ibaraki, Japan	–
H1	Os17	*Oryza sativa* L.	Hitachi, Ibaraki, Japan	–
H1	St29	*Solanum tuberosum* L.	Ibaraki, Ibaraki, Japan	–
H2	Af79	*Allium fistulosum* L.	Takahagi, Ibaraki, Japan	–
H2	Boc86	*Brassica oleracea* var. *capitata* L.	Hitachiota, Ibaraki, Japan	–
H2	Brl5	*Brassica rapa* var. *laciniifolia*	Hitachiota, Ibaraki, Japan	–
H2	Brn1	*Brassica rapa* var. *nippo-oleifera*	Hitachiota, Ibaraki, Japan	–
H2	Brn9	*Brassica rapa* var. *nippo-oleifera*	Hitachiota, Ibaraki, Japan	+
H2	Ls9	*Lactuca sativa* L.	Tsukuba, Ibaraki, Japan	–
H2	Pas1	*Plantago asiatica*	Fujimi, Nagano, Japan	–
H2	St290	*Solanum tuberosum* L.	Atsugi, Kanagawa, Japan	–
H2	Vf3	*Vicia faba* L.	Hitachiota, Ibaraki, Japan	–
H3	St367	*Solanum tuberosum* L.	Kiyosato, Hokkaido, Japan	–
H3	St386	*Solanum tuberosum* L.	Honbetsu, Hokkaido, Japan	–
H4	St316	*Solanum tuberosum* L.	Nakasatsunai, Hokkaido, Japan	–
H5	Cab53	*Capsella bursa-pastoris* (L.) Medik.	Honbetsu, Hokkaido, Japan	–
H5	Pas29	*Plantago asiatica*	Hitachi, Ibaraki, Japan	–
H5	St528	*Solanum tuberosum* L.	Shimabara, Nagasaki, Japan	–
H5	Tre5	*Trifolium repens* L.	Kutsukake, Toyoake, Aichi, Japan	–
H6	Seg1	*Setaria glanea*	Fujimi, Nagano, Japan	–
H7	Ost2	*Oenothera stricta* Ledeb.	Fujimi, Nagano, Japan	+
H8	Pc102	*Phaseolus coccineus* L.	Tsukuba, Ibaraki, Japan	–
H8	Sm6	*Solanum melongena* L.	Sakae, Toyoake, Aichi, Japan	–

[a] For the biocontrol activities against cabbage damping-off caused by *Rhizoctonia solani*, the previously described data (Someya *et al.*, 2020) were used with modification. +++, <75% inhibition; ++, 50%–75% inhibition; +, 25%–50% inhibition; –, 0%–25% inhibition.

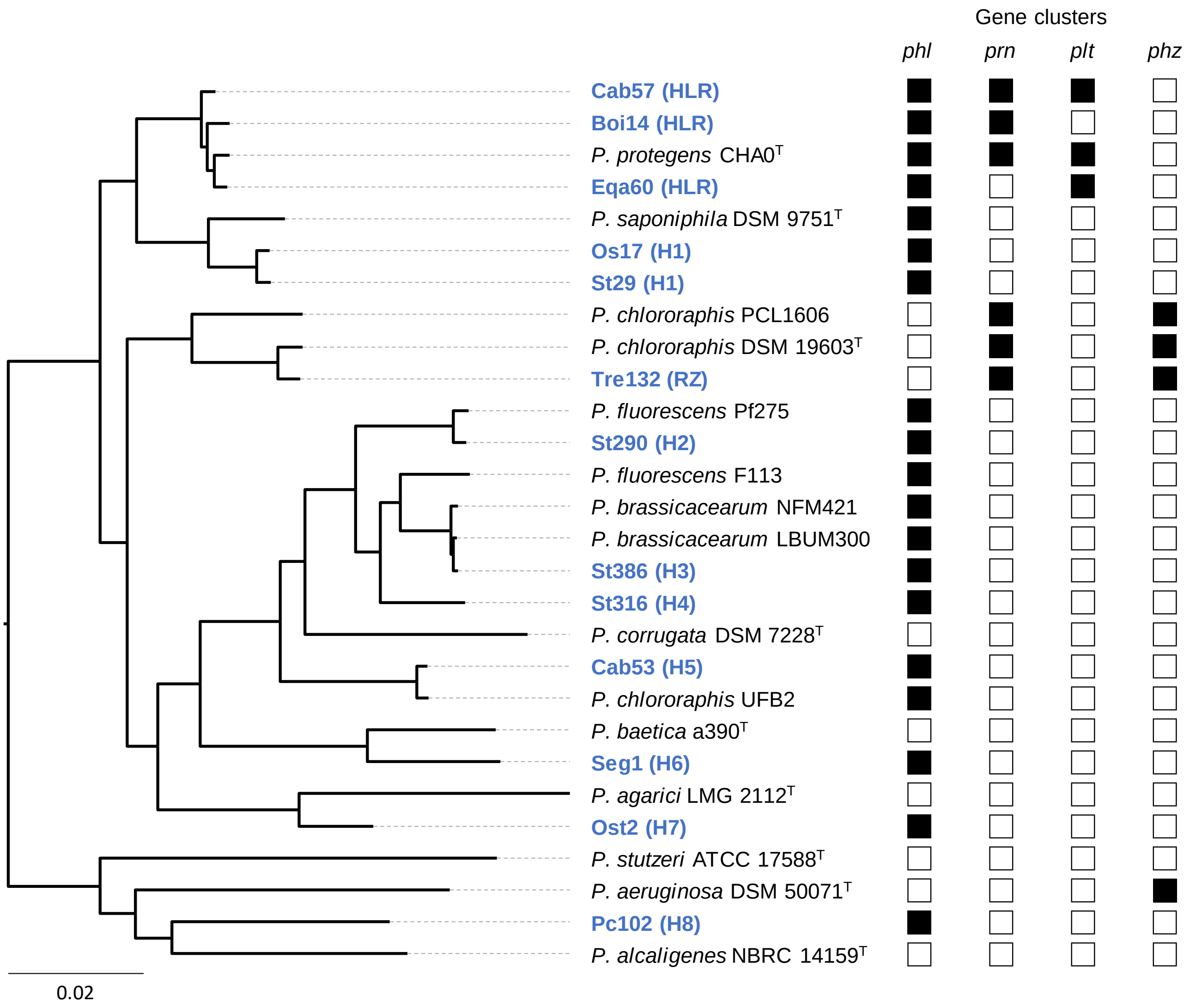

Fig. S1. Phylogenetic tree based on the complete sequences of fluorescent pseudomonads. The phylogenetic tree was constructed by REALPHY 1.12. Strains whose genome was sequenced in our present or previous study are depicted in blue. The presence (filled square) or absence (open square) of the *phl*, *prn*, *plt*, and *phz* gene cluster are described on the right side of the tree.

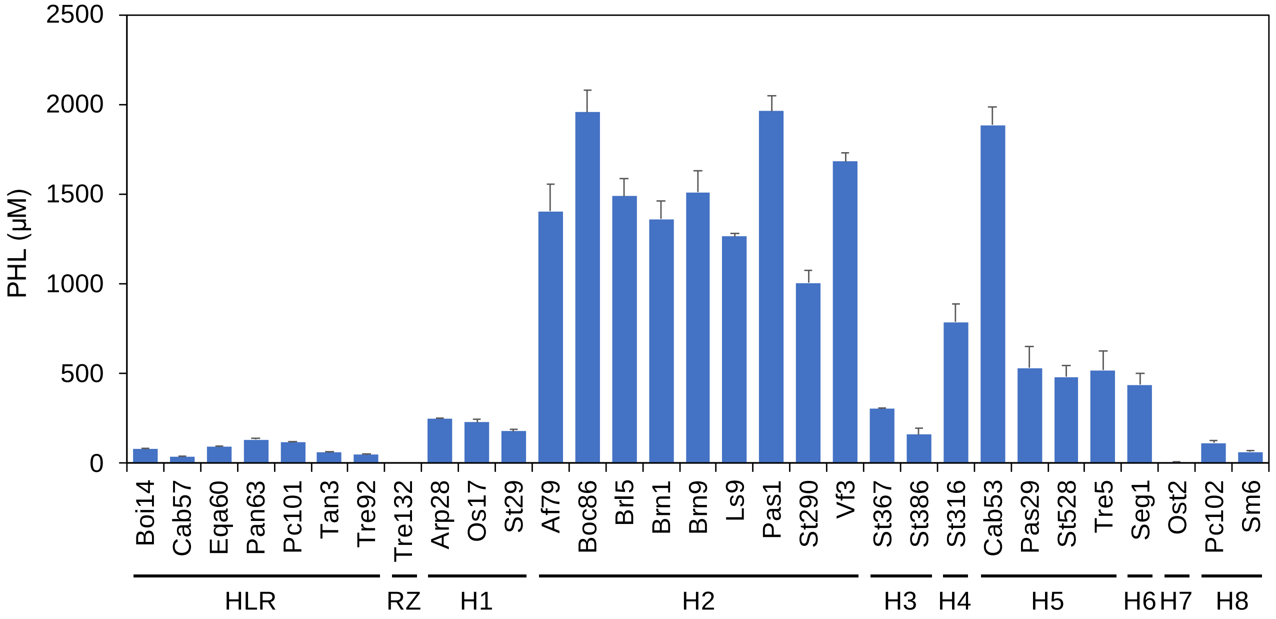

Fig. S2. PHL production of fluorescent pseudomonads harboring the phl gene cluster. Details of strains used in this assay are described in Table S1. The data were reproduced at least three times, and error bars indicate standard deviations.
